# Impact of a probiotic fermented milk in the gut ecosystem and in the systemic immunity using a non-severe protein-energy-malnutrition model in mice

**DOI:** 10.1186/1471-230X-11-64

**Published:** 2011-05-26

**Authors:** Carolina Maldonado Galdeano, Ivanna Novotny Núñez, Alejandra de Moreno de LeBlanc, Esteban Carmuega, Ricardo Weill, Gabriela Perdigón

**Affiliations:** 1 Centro de Referencia para Lactobacilos (CERELA-CONICET). Chacabuco 145, San Miguel de Tucumán (T4000ILC) Tucumán. Argentina; 2 Cátedra de Inmunología. Instituto de Microbiología. Facultad de Bioquímica, Química y Farmacia. Universidad Nacional de Tucumán, Argentina; 3 Nutritia. Buenos Aires. Argentina; 4 Departamento de Investigación y Desarrollo, DANONE Argentina S.A. Buenos Aires. Argentina

## Abstract

**Background:**

Malnutrition affects the immune response, causing a decrease of defence mechanisms and making the host more susceptible to infections. Probiotics can reconstitute the intestinal mucosa and stimulate local and systemic immunity. The aim of this work was evaluate the effects of a probiotic fermented milk as a complement of a re-nutrition diet, on the recovery of the intestinal barrier, and mucosal and systemic immune functions in a murine model of non-severe protein-energy-malnutrition. Its potential protection against *Salmonella enterica *serovar Typhimurium (*S*. Typhimurium) infection was also analyzed.

**Methods:**

Mice were undernourished and divided into 3 groups according to the dietary supplement received during re-nutrition (milk, probiotic fermented milk or its bacterial free supernatant) and compared to well-nourished and malnourished mice. They were sacrificed previous to the re-nutrition and 5 days post re-nutrition. The phagocytic activity of macrophages from spleen and peritoneum and the changes in the intestinal histology and microbiota were evaluated. Different immune cell populations and cytokine productions were analyzed in the small intestine tissues. The effect of the re-nutrition supplements on the systemic immunity using OVA antigen and against an infection with *S. *Typhimurium was also studied.

**Results:**

Probiotic fermented milk was the most effective re-nutrition diet that improved the intestinal microbiota. Its administration also increased the number of IgA+ cells, macrophages and dendritic cells. The production of different cytokine (IFN-γ, TNF-α, IL-12) by these cells and the phagocytic activity in peritoneum and spleen was also increased. This re-nutrition diet also stimulated the systemic immune response against OVA antigen which was diminished after the malnutrition period and also improved the host response against *S. *Typhimurium, decreasing the spread of pathogenic bacteria to the liver and the spleen. The importance of the metabolites released during milk fermentation was also demonstrated through the analysis of the bacterial free supernatant obtained from the probiotic fermented milk, but the whole product showed the best effects in the parameters evaluated in this study.

**Conclusions:**

The administration of probiotic fermented milk as a dietary supplement during the re-nutrition process in a murine immunodeficiency model by malnutrition could be a good adjuvant diet to improve the gut and systemic immune response for the protection against *Salmonella *infection.

## Background

Malnutrition is a systemic alteration, potentially reversible, caused by imbalance between the nutrient intake and energy requirements [1]. It is related mainly with decrease of the growth and development, reduced capacity for learning and depression of the immune system [2]. It is known that deficiencies as well as excesses of nutrients can negatively affect the number and activity of immune cells [3,4]. The function of the immune cells diminishes according to the severity of the malnutrition, which depends on the degree of nutrients imbalance, the interaction between nutrients and the age of the host [5].

The protein-energy malnutrition (PEM) is classified according to weight loss in: slight (10-25%), moderate (25%-39%) and severe (> 40%). Their consequences include loss of weight and delay in the growth, often associated with diverse clinical syndromes accompanied by mineral or vitamin deficiencies and immune deficiencies. The PEM can be reversed with an appropriate re-nutrition, which can restore all gastrointestinal and immune functions, regenerate the intestinal mucosa, and improve its microbiota.

Probiotics are defined as live microorganisms which, when administered in adequate amounts confer a health benefit on the host [6]. It is known that probiotics in healthy hosts can reconstitute the intestinal mucosa through the reduction of its permeability and can strengthen the local immune response, particularly through the IgA and systemic immunity by acting as an adjuvant [7]. These microorganisms can also influence the composition and activity of the gut microbiota, modulate the inflammatory response and improve the non-specific intestinal barrier [8].

In this sense, probiotic administration as a re-nutrition complement could be useful in malnourished groups prior to vaccination due to the adjuvant properties attributed to them.

The beneficial effect of yogurt and probiotic bacteria in malnutrition has been shown in previous studies using a mouse model of severe PEM [9] as well as in animal models fed with high energy diet [10].

The aim of the present work was to evaluate the effects of a probiotic fermented milk (PFM) as a complement of a re-nutrition diet, on the recovery of the intestinal barrier, histological alterations, the mucosal and systemic immune functions and its protective effect against *Salmonella enterica *serovar Typhimurium (*S*. Typhimurium) infection, using a model of non-severe PEM in mouse. The effects obtained with the PFM were also compared to those obtained with non-fat milk or with the bacterial free supernatant of the PFM to evaluate the contribution of biological active components produced during fermentation such as peptides and carbohydrates, and natural components presents in the milk, on the immune system recovery.

## Methods

### Animals. Malnutrition and re-nutrition protocols

BALB/c mice were obtained from the closed random bred colony maintained at the CERELA (Centro de Referencia para Lactobacilos, San Miguel de Tucumán, Argentina). After weaning (21 days), the mice were divided in two control groups and three test groups: **Well nourished control (WC)**, ten animals that received conventional balanced diet (23% proteins, 6% raw fibre, 10% total minerals, 1.3% Ca, 0.8% P, 12% moisture and vitamins). **Malnourished Control (MC)**, twenty mice fed during 5 days with conventional diet, without restriction. After that, the malnourishment period started and the animals received restricted food (25% less than the WC group) [11]. These animals were not re-nourished and continued with restricted food intakes during all the experiment. Mice of these two groups received water *ad-libitum*.

Mice of the three test groups (fifteen for each group) received the restricted diet as was explained for MC group and when the animals lost 25% of body weight compared to WC, they were re-nourished during 5 days, due to this time was the optimal period for the PFM to activate the intestinal mucosa immune system in healthy animals [12], with three different dietary supplements: **Milk (M)**, mice were re-nourished with 10% whole milk. **PFM**, mice were re-nourished with a commercial fermented milk containing the yogurt starter cultures (*Lactobacillus *(*L*.) *delbrueckii *subsp. *bulgaricus *10^8 ^CFU/ml and *Streptococcus thermophilus *10^8 ^CFU/ml) and the probiotic bacterium *L. casei *DN-114-001 (10^8 ^CFU/ml). **Bacterial free supernatant (BFS): **mice were re-nourished with a bacterial free supernatant, obtained by centrifugation of PFM at 10000 *g *during 20 min. The supernatant was plated in MRS agar and the bacterial count was lower than 10^2 ^CFU/ml.

**Figure 1:**
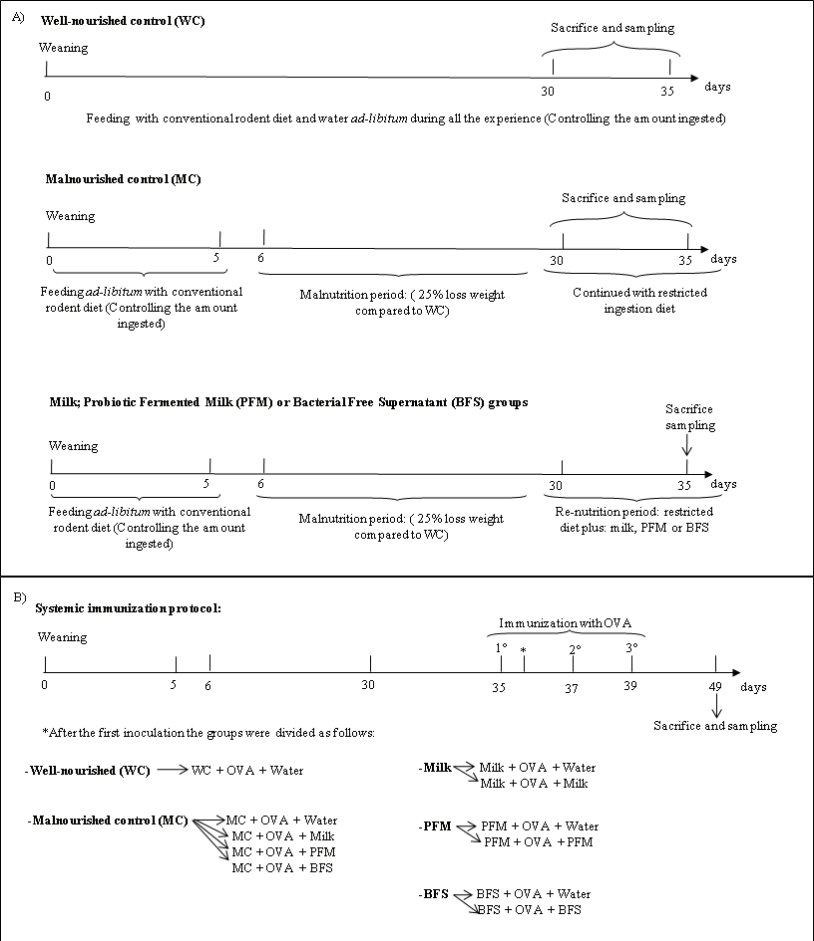
**Design of the different experimental groups under study**. A) Protocol for malnutrition and re-nutrition with the different dietary supplements. Mice were sacrificed at day 30 (basal sample) and 5 days after re-nutrition (day 35). B) Protocol for systemic immunization. 1^st^, 2^nd ^and 3^rd ^indicate the day of the OVA injections. Arrow shows the day of the sacrifice (10 days after the last immunization, day 49).

Mice of the three test groups received the specific diet supplement and water *ad-libitum*, maintained the restriction of the conventional diet (25% of the WC ingest) during 15 days, in a room with a 12-h light/dark cycle at 18 ± 2°C. They were weighed daily and three mice per group were sacrificed by cervical dislocation at 0 (basal sample) and 5 days after re-nutrition. Samples of small and large intestines were obtained for immunological and microbiological studies. Macrophages were also removed from peritoneum and spleen to evaluate the phagocytic activity (Figure 1A). All animal protocols were preapproved by the Animal Protection Committee of CERELA and all experiments comply with the current laws of Argentina.

### Systemic immunization protocol

To evaluate the systemic immunity, animals were divided in 5 experimental groups: 3 from WC, 12 from MC and 6 re-nourished animals for each test group. Test groups received during 5 days re-nutrition diet supplemented with PFM, milk or BFS; after that all groups of animals (controls and tests) were injected subcutaneously, three times every 48 hours, with 15 μg of chicken egg albumin (OVA) in phosphate buffered solution (PBS). After the first injection, each test group was subdivided in two groups: 1) three mice that stopped the complementary diet to study the effect of the administration of each treatment prior to the immunization, and 2) three mice that continued receiving the corresponding supplement diet during and after immunization until the end of the experiment (10 days post immunization) to evaluate if the continuous administration increased its adjuvant effect on the anti-OVA response. Simultaneously, the MC group was divided in four groups of 3 mice each one: 1) mice that continued with the restricted diet and water (malnourished control) and 2), 3), and 4) mice that received milk, PFM or BFS, respectively to evaluate the adjuvant effect of the different supplementary diets during and post immunization, as was explained above (Figure 1B describes the experimental design). Three mice per group were sacrificed 10 days after the last injection (day 49). Blood was collected to determine specific anti-OVA IgG in the serum by ELISA test. At this time point peritoneal and spleen macrophages were isolated to study the phagocytic activities.

### Colonization assays

To analyze possible secondary effects of re-nutrition, such as bacteria translocation from intestine to other organs, animals from each test group were sacrificed after 5 days of re-nutrition with the different diets, and the livers were aseptically removed, weighed and placed into sterile tubes containing 5 ml of peptone water (0.1%). The samples were immediately homogenized under sterile conditions using a microhomogenizer (MSE, England). Serial dilutions were made and spread onto the surface of MacConkey and MRS agar (Britania, Buenos Aires, Argentina). The plates were then incubated aerobically at 37°C for 24 or 48 h, for each medium respectively.

### Analysis of the intestinal microbiota

The large intestines were aseptically removed after 5 days of re-nutrition, weighed and placed into sterile tubes containing 5 ml of peptone water (0.1%). The samples were immediately homogenized under sterile conditions. Serial dilutions of the homogenized samples were obtained and aliquots (0.1 ml) of the appropriate dilution were spread onto the surface of following agarized media: Reinforced Clostridial agar (RCA, Britania, Buenos Aires, Argentina) for total anaerobic bacteria; RCA containing 0.2% LiCl, colistin 4 mg/l, 1% aniline blue and after sterilization adjusted to pH 5 with acetic acid (RCA-pH5) for isolation of bifidobacteria; Mann-Rogosa-Sharp (MRS Britania, Buenos Aires, Argentina) for total lactobacilli; and MacConkey (Britania, Buenos Aires, Argentina) for Enterobacteriaceae. MacConkey and MRS agar were aerobically incubated at 37°C for 24 h and 48 h, respectively, and the others cultures media were anaerobically incubated at 37°C for 72-96 h.

### Histological samples

The small intestines were also removed after 5 days of re-nutrition and washed with saline solution (NaCl 0.15 M). The tissues were prepared using the method described by Sainte-Marie (1962). Serial paraffin sections (4 μm) were made and used for haematoxylin eosin staining and immunofluorescence assays to determine different immunological markers to characterized T populations, macrophages (MQ) and dendritic cells (DC) as well as the IgA and cytokine positive cells in the lamina propria of the intestine.

### Immunofluorescence assay for IgA-secreting cells, CD4+ and CD8+ T lymphocytes

The number of IgA+ cells, CD4+ and CD8+ T lymphocytes were determined by direct immunofluorescence assays. After deparaffinization using xylene and rehydration in a decreasing gradient of ethanol, slides were incubated with α-chain monospecific antibody conjugated with fluorescein isothiocyanate (FITC, Sigma, St. Louis, USA) for IgA+ cells or with monoclonal antibodies conjugated with FITC (Cedarlane, Ottawa, Canada) for CD4+ or CD8+ T lymphocytes. The results are expressed as the number of positive cells per 10 fields of vision (magnification 1000×) using a fluorescent light microscope.

### Determination of macrophage and dendritic cells

Macrophages and DC were determined using indirect immunoflourescence in lamina propria of the small intestine tissues as was described previously [12]. The BM8 monoclonal antibody (Affinity Purified anti-mouse F4/80 Antigen - Pan Macrophage Marker, eBioscience, San Diego, CA, USA) was used for macrophages and, the 33D1 monoclonal antibody (Affinity Purified anti-mouse Dendritic Cell Marker (33D1) eBioscience, San Diego, CA, USA), which recognizes a mouse dendritic cell-specific surface marker. The results were expressed as the number of positive fluorescent cells per ten fields of vision (1000X).

### Immunohystochemical detection of cytokine-producing cells

Cytokine-producing cells were also detected in lamina propria of the small intestine by indirect immunofluorescence assay following the technique described by Perdigón et al. [13]. The rabbit anti-mouse TNF-α, IFN-γ, IL-10, IL-6, IL-2, and IL-4 (Peprotech, Inc. Rocky Hill, NJ, USA) polyclonal antibodies and goat anti-mouse IL-12 antibody were used. The results were expressed as the number of positive fluorescent cells per ten fields of vision (1000X).

### Determination of goblet cells

To determine whether or not the dietary supplement had influence on the nonspecific barrier, the number of goblet cells was studied. Slides from the small intestine from the different groups were deparaffinised and rehydrated in a decreasing gradient of ethanol and incubated in 1% Alcian Blue 8Gx solution (Merck, Darmstadt, F.R. Germany) in 3% acetic acid. Histological slides were incubated in eosin solution and then in 0.5% safranin solution in 0.1 N HCl. They were then dehydrated and finally mounted using synthetic Canada balsam (Ciccarelli Lab., San Lorenzo, Argentina). Goblet cells were stained blue with this methodology. The results are expressed as the number of goblet cells per ten intestinal villous.

### Determination of specific anti-OVA IgG

The ELISA test was performed using 96 wells microplates that were coated with 1% w/v of OVA solution in carbonate buffer (pH 9.5) and incubated overnight at 4°C. Non-specific protein-binding sites were blocked with PBS containing 0.5% non-fat milk (PBS-milk). Dilutions of the test and control serum samples were placed into the PBS-milk and then incubated at room temperature for 2 h. After washing with PBS containing 0.05% Tween 20 (PBS-T), the plates were incubated 1 h with biotin-SP-conjugated goat anti-mouse IgG specific antibody (Jackson Immuno Research Labs Inc, West Grove, USA) and then they were incubated with enzyme concentrated avidin - HRP (BD Bioscience Pharmingen, San Diego, USA) during 30 min. The plates were again washed and the TMB reagent (3,3', 5,5' tetramethylbenzidine, BD Biosciences, San Diego, USA) was added and incubated during 20 min. The reaction was stopped with H_2_SO_4 _(2 N). The absorbance was read at 450 nm and the results are expressed as OD.

### Ex vivo phagocytosis assay of peritoneal and spleen macrophages

Peritoneal and spleen macrophages from the three time points: 0 (basal sample), 5 days of re-nutrition (previous to the immunization) and 10 days post immunization were obtained according to de Moreno de LeBlanc et al. [14] and the concentration were adjusted at 1 × 10^6 ^cells/ml. Phagocytosis assay was performed using a *Saccharomyces boulardii *suspension at a concentration of 10^7 ^cells/ml. Opsonized yeast in mouse autologous serum (10%) were added to 0.2 ml of macrophage suspension. The mixture was incubated for 30 min at 37°C. The percentage of phagocytosis was expressed as the percentage of phagocyting macrophages in 200 cells counted in an optical microscope.

### *Salmonella enterica *serovar Typhimurium infection

For this assay, after 5 days of re-nutrition with the different dietary supplements, ten mice from each group were challenged intragastrically with 100 μl/mice of *S. *Typhimurium (at a concentration of 1 × 10^8 ^cells/ml). Three animals per group were sacrificed 4 days post-challenge, and spleen, liver and large intestine were aseptically removed, weighed and placed into sterile tubes containing 5 ml of peptone water (0.1%). The samples were immediately homogenized and serial dilutions were made and spread onto the surface of MacConkey agar for spleen and liver and Salmonella-Shigella agar (Britania, Buenos Aires, Argentina) for large intestine. The plates were then incubated aerobically at 37°C for 24 h.

### Statistical analysis

Statistical analysis were performed using MINITAB 14 software (Minitab, Inc., State College, PA, USA) by ANOVA GLM followed by a Tukey's posthoc test, and *P *< 0.05 was considered significant. Unless otherwise indicated, all values (n = 9) were the means of 3 independent trials (no significant differences were observed between individual replicates) ± standard deviation (SD).

## Results

### Effect of milk, PFM or BFS supplementation on body weight, intestinal microbiota and translocation to the liver

**Figure 2:**
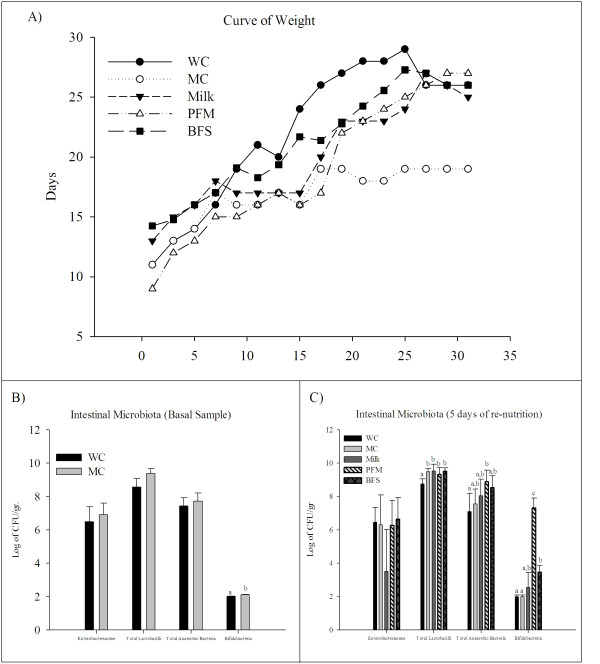
**Changes in the body weight and the intestinal microbiota of the large intestine**. Weight changes of WC, MC groups and mice re-nourished with milk, PFM and BFS. Each point represents average ± SD of weight data pooled from 30 mice (10 mice from each separated trial). The large intestine were aseptically removed, weighed and placed into sterile tubes containing peptone water and aliquots of the appropriate dilution were spread onto the surface of different agarized media. Results are expressed as means ± SD of the log_10 _CFU/g large intestine. A) Represents the basal data, after the malnutrition period, comparing MC and WC groups. B) Represents the results obtained after 5 days of re-nutrition (day 35) in all the groups assayed. Each mean represents data from nine animals. ^a,b,c^Means for each medium without a common letter differ significantly (p < 0.05).

The results showed that the weight in the mice of MC group decreased significantly compared to WC group. The animals that were re-nourished with milk, PFM or BFS showed body weight gain compared to MC group (Figure 2A). The values obtained from all test groups were near to WC group.

The study of the intestinal microbiota showed a significant increase of bifidobacteria count in mice re-nourished with PFM compared to the other groups. An increase in the bifidobacteria population was also observed in the mice given BFS compared to both control groups (Figure 2B and 2C). No significant differences were observed for the other bacteria populations between the MC and the test groups. Total *lactobacilli *increased in MC mice compared to WC group, and different re-nutrition diets maintained this increase without significant differences with MC group (Figure 2C).

The analysis of the bacterial translocation to the liver in the malnourished mice showed increased bacterial count in MRS medium. The three re-nutrition diet prevented bacterial translocation to the liver (data not shown).

### Effect of milk, PFM or BFS on the histology of the small intestine

**Figure 3:**
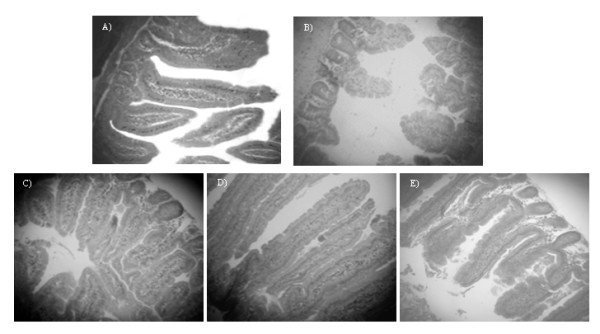
**Histological study comparing the different experimental groups**. Slices from small intestine of mice were studied after staining with hematoxilin-eosin. All the samples were obtained after 5 days of re-nutrition with a magnification of 100×. A) WC group B) MC group: the intestinal villi are short and their numbers are lower than in the WC group. The three re-nutrition dietary supplements: milk (C), PFM (D) and BFS (E) improved the histological alterations caused by malnutrition, being the mice that received PFM, the group that showed histological characteristics similar to the WC animals.

The histology of the small intestine was affected in MC group, the intestinal villi were shorter and their numbers were lower than in the WC group (Figure 3A and 3B). The administration of the PFM during the re-nutrition period significantly improved these histological alterations (Figure 3C). The mice that received milk or BFS also showed the same effect, but the improvement was lower than in the animals that received PFM (Figure 3D and 3E).

### Effect on the goblet cells, IgA secreting cells and T lymphocytes

All the test groups showed similar values for goblet cells without significant differences compared to both control groups, showing that the non severe malnutrition in our experimental model does not affect the number of these cells (Table 1).

**Table 1:** IgA-secreting cells, goblet cells and T lymphocytes on the small intestine

Sample	Group	IgA+ cells	Goblet cells	CD4+ TL	CD8+ TL
Basal	WC	191 ± 36 ^a^	46 ± 14 ^a^	43 ± 11 ^a^	30 ± 9 ^a,b^
	MC	147 ± 44 ^a,b^	42 ± 23 ^a^	23 ± 8 ^b^	17 ± 6 ^a^

5 days of re-nutrition	WC	143 ± 26 ^a,b^	51 ± 13 ^a^	47 ± 9 ^a^	40 ± 7 ^b^
	MC	106 ± 33 ^b^	41 ± 14 ^a^	30 ± 7 ^b,c^	24 ± 7 ^a^
	Milk	100 ± 32 ^b^	48 ± 27 ^a^	53 ± 17 ^a,c^	26 ± 10 ^a,b^
	PFM	163 ± 31 ^a,b^	34 ± 9 ^a^	36 ± 9 ^a,b^	20 ± 5 ^a^
	BFS	124 ± 39 ^a,b^	32 ± 8 ^a^	28 ± 8 ^a,b^	24 ± 8 ^a^

The number of IgA+ cells, CD4+ and CD8+ T lymphocytes were determined by direct immunofluorescence on the small intestine tissue slides of mice from different experimental groups (well-nourished control (WC), malnourished control (MC) and re-nourished with milk, probiotic fermented milk (PFM) or bacterial free supernatant (BFS)). The results were expressed as the number of positive cells per ten fields of vision (1000X). Goblet cells were stained with alcian blue on slides from the small intestine of mice from different groups under study and the results were expressed as number of positive cells per 10 villi. Values are means for n = 9 ± SD mice from each group and sample. ^a,b,c ^Means for each cell population without a common letter differ significantly (*P *< 0.05).

The number of IgA+ cells decreased in MC group, but the mean value was not significantly diminished compared to WC group in the basal sample, before the re-nutrition period (191 ± 36 and 147 ± 44, respectively). Five days of PFM administration as re-nutrition diet significantly increased the number of IgA+ cells (163 ± 25) compared to the mice from MC group (106 ± 28). The other re-nutrition diets did not increase these cells, maintained their numbers similar to the MC group (Table 1).

The number of both CD4+ and CD8+ cells of the malnourished mice decreased significantly (23 ± 8 and 30 ± 7; 17 ± 6 and 24 ± 7, respectively), compared to WC mice (43 ± 11 and 47 ± 9; 30 ± 9 and 40 ± 7, respectively) in the basal sample and 5 days after that. No significant differences were observed for the groups fed 5 days with milk, PFM or BFS compared to MC group (Table 1).

### Influence on marker expression in macrophages and DC

Macrophages were identified using the BM8 monoclonal antibody which reacts with mouse F4/80 antigen. The analysis of macrophages showed that this cell population decreased significantly after the malnutrition period (74 ± 22 and 83 ± 11) compared to WC (141 ± 20 and 122 ± 24) at the basal sample and 5 days after that, respectively (Table 2). The three re-nutrition diets increased the number of macrophages reaching values similar to the WC.

**Table 2:** Macrophages and dendritic cells in the lamina propria of the small intestine

Sample	Group	Macrophages	Dendritic cells
Basal	WC	141 ± 20 ^a^	64 ± 17 ^a,d^
	MC	74 ± 22 ^b,c^	27 ± 3 ^b^

5 days of re-nutrition	WC	122 ± 24 ^a,d^	40 ± 9 ^a,c^
	MC	83 ± 11 ^b^	16 ± 6 ^b^
	Milk	114 ± 19 ^a,c,d^	26 ± 11 ^b,c^
	PFM	116 ± 14 ^a,d^	57 ± 19 ^a,d^
	BFS	101 ± 07 ^c,d^	80 ± 19 ^d^

Macrophages and dendritic cells were determined by indirect immunofluorescence on the small intestine tissue slides of mice from different experimental groups (well-nourished control (WC), malnourished control (MC), and re-nourished with milk, probiotic fermented milk (PFM) or bacterial free supernatant (BFS)).

Results were expressed as number of positive cells recognized for the respective primary antibody, counted in 10 fields of vision at 1000× of magnification. Values are means for n = 9 ± SD mice from each group and sample. ^a,b,c,d^Means for each cell population without a common letter differ significantly (*P *< 0.05).

DC were determined using the 33D1 monoclonal antibody which recognizes a mouse DC-specific surface marker. These cells decreased significantly in malnourished mice (27 ± 3 and 15 ± 6) compared to WC mice (64 ± 17 and 40 ± 9) at both time points assayed, respectively. Re-nutrition with PFM and BFS recovered significantly the numbers of DC that were similar or higher than those obtained in the WC group. Milk administration did not increase the number of DC (Table 2).

### Determination of cytokine expression

IL-2 and IL-12 decreased in MC group compared to WC. Both IL-2 and IL-12+ cells increased significantly in the small intestine of mice re-nourished with milk (47 ± 11 and 42 ± 12), and PFM (37 ± 10 and 36 ± 11) compared to the MC (20 ± 7 and 16 ± 5). BFS administration increased significantly the number of IL-2+ cells (34 ± 6), but no significant differences were observed for IL-12+ cells (21 ± 5) compared to MC group (Table 3).

**Table 3:** Cytokine-producing cells in the lamina propria of the small intestine

Sample	Groups	IL-2	IL-12	TNF-α	IFN-γ	IL-6	IL-4	IL-10
Basal	WC	27 ± 8 ^a^	29 ± 8 ^a,b^	41 ± 12 ^a^	31 ± 8 ^a, b^	26 ± 5 ^a^	36 ± 10 ^a^	15 ± 4 ^a^
	MC	16 ± 4 ^b^	22 ± 8 ^a,b^	21 ± 7 ^b^	20 ± 6 ^a^	16 ± 3 ^b,c^	19 ± 6 ^b,c^	8 ± 2 ^b^

5 days of re-nutrition	WC	31 ± 9 ^a^	32 ± 12 ^a,b^	52 ± 14 ^a^	33 ± 13 ^a,b^	25 ± 7 ^a,b^	34 ± 9 ^a,b^	19 ± 6 ^a^
	MC	20 ± 7 ^a,b^	16 ± 5 ^a^	21 ± 6 ^b^	20 ± 5 ^a^	13 ± 6 ^b,c^	18 ± 5 ^c^	8 ± 2 ^b^
	Milk	47 ± 11 ^a^	42 ± 12 ^b^	39 ± 6 ^a^	43 ± 8 ^b^	19 ± 3 ^a,b,c^	91 ± 19 ^d^	20 ± 5 ^a^
	PFM	37 ± 10 ^a^	36 ± 11 ^b,c^	41 ± 8 ^a^	39 ± 10 ^b,c^	14 ± 3 ^c^	38 ± 10 ^a^	10 ± 4 ^a,b^
	BFS	34 ± 6 ^a^	21 ± 5 ^a,c^	17 ± 4 ^b^	27 ± 6 ^a,c^	24 ± 4 ^a^	27 ± 9 ^a,b,c^	8 ± 3 ^a,b^

Cytokine-producing cells were detected in the lamina propria of the small intestine of mice from different experimental groups (well-nourished control (WC), malnourished control (MC), and re-nourished with milk, probiotic fermented milk (PFM) or bacterial free supernatant (BFS)) by indirect immunofluorescence assay. The rabbit anti-mouse TNF-α, IFN-γ, IL-10, IL-6, IL-2, and IL-4 polyclonal antibodies and goat anti-mouse IL-12 antibody were used. Results are expressed as the number of positive fluorescent cells, counted in 10 fields of vision at 1000× of magnification. Values are means for n = 9 ± SD mice from each group and sample. ^a,b,c,d^Means for each cell population without a common letter differ significantly (*P *< 0.05).

Re-nutrition with milk or PFM increased significantly the number of IFN-γ+ cells (43 ± 8 and 39 ± 10) and TNF-α+ cells (39 ± 6 and 41 ± 8) compared to the MC group (20 ± 5 and 21 ± 6 for IFN-γ and TNF-α, respectively). Mice administered BFS maintained similar values for both cytokines to those of MC group (Table 3).

The analysis of IL-6+ cells showed that only the mice given BFS increased significantly these cells (24 ± 4) compared to MC group (13 ± 6), reaching the numbers observed in the WC mice (25 ± 7, Table 3).

IL-4 and IL-10+ cells had a similar behaviour, only the group of mice that received milk increased significantly the numbers of both cytokine secreting cells (91 ± 19 and 20 ± 5, respectively) compared to MC group (18 ± 5 and 8 ± 3, respectively, Table 3).

### Determination of the phagocytic activity of macrophages isolated from spleen and peritoneum before and after OVA immunization

The percentage of phagocytic activity of spleen and peritoneal macrophages increased significantly after 5 days of re-nutrition in the mice that received PFM (20 ± 6 and 26 ± 1) and BFS (31 ± 8 and 28 ± 3) compared to MC group (14 ± 4 and 18 ± 4) for spleen and peritoneum, respectively. The group re-nourished with milk only increased phagocytosis in the macrophages isolated from spleen (31 ± 8), compared to MC group.

**Figure 4:**
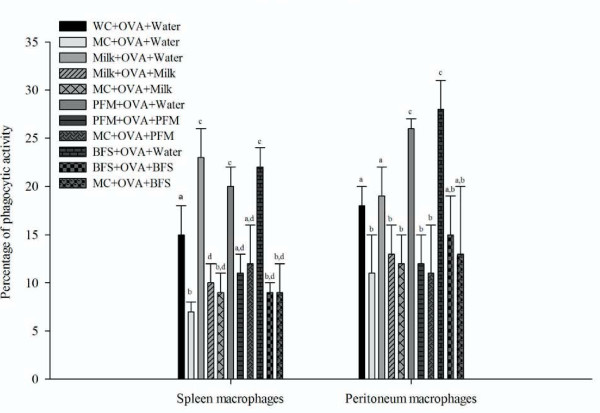
**Phagocytic activity of macrophages isolated from spleen and peritoneum**. Mice were sacrificed 10 days after the 3^rd ^OVA injection to isolate the macrophages from peritoneum and spleen. The activity of these cells was determined by phagocytosis assay using *Saccharomyces boulardii*. The values are expressed as mean for N = 9 ± SD of percentage of phagocytosis expressed as the percentage of phagocyting macrophages in 200 cells counted. ^a,b,c,d^Means values for spleen or peritoneum without a common letter differ significantly (P < 0.05).

The assay performed in mice immunized with OVA showed that in the sample taken 10 days after last OVA injection; MC group maintained the decrease of the phagocytic activity. The administration of any re-nutrition supplement diet during 5 days previous to the immunization increased significantly the percentage of phagocytic activity of spleen and peritoneal macrophages compared to MC, WC and the rest of the test groups. Mice that received continuous administration of supplementary diet under study (before and after OVA immunization) did not show significant differences compared to MC mice that received the same supplementary diet during and after OVA immunization (MC-OVA-Milk, MC-OVA-BFS and MC-OVA-PFM (Figure 4).

### Effect on systemic antibody response

Specific anti-OVA IgG increased significantly in the serum of mice from the three test groups when they were administered continuously (before and after OVA immunization) with milk (0.94 ± 0.2), PFM (1.16 ± 0.05) and BFS (0.94 ± 0.1) compared to the MC (0.4 ± 0.3).

**Figure 5:**
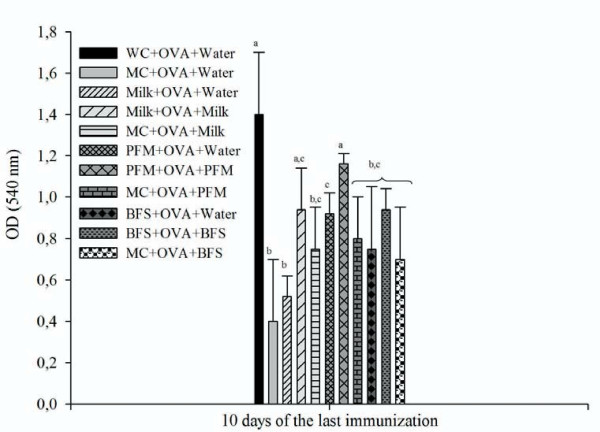
**Specific anti-OVA IgG determination in blood serum**. ELISA was used to measure the concentration of anti-OVA IgG in the blood serum obtained from mice of different experimental groups 10 days after the 3^rd ^OVA injection. Results are expressed as OD (450 nm). Each point represents the mean of n = 9 ± SD mice from each group. ^a,b,c,^Means values without a common letter differ significantly (P < 0.05).

Specific anti-OVA IgG in serum also increased in the group of mice given PFM previous to the immunization (0.92 ± 0.1, Figure 5).

### Effect of PFM in malnourished mice challenged with *S. *Typhimurium

**Figure 6:**
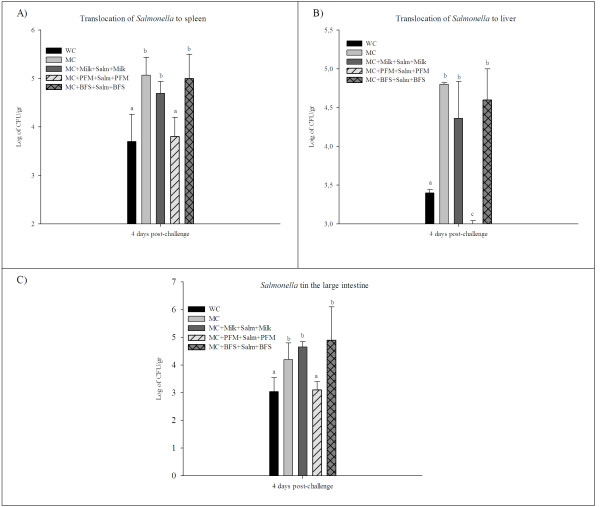
***S. *Typhimurim counts in the large intestine and bacterial translocation to spleen and liver**. Mice were infected with *S. *Typhimurim at day 35 of the experimental protocol. They were sacrificed 4 days after challenged and the spleen, liver and large intestine were collected for the determination of the pathogen in agarized media. Colony counts are expressed as log_10 _numbers of bacteria per gram of organ. Each value represents the mean of N = 9 ± SD. ^a,b,c^For each organ means without a common letter differ significantly (p < 0.05).

The study of the *S. *Typhimurium translocation to spleen, liver and large intestine, showed a significant increase in MC mice compared to the WC mice. The animals re-nourished with PFM showed a significant decrease significantly of *Salmonella *counts in the spleen and large intestine compared to MC group, without significant differences with WC mice. The translocation to the liver in the mice re-nourished with PFM was even significantly lower than in the WC group. No protective effect was observed in the mice that received the others re-nutrition diets (Figure 6A, B and 6C).

## Discussion

The administration of PFM or its BFS as a re-nutrition supplementation in a non-severe PEM model caused a weight gain in the animals, being the BFS which showed the fastest effect (after 15 days, Figure 2A). Considering that the intestinal architecture is altered during malnutrition state [7] and that PFM or its BFS administration improved the gut histology faster than milk alone (Figure 3), the microbial translocation to liver was analyzed. It was observed that increased bacterial translocation in the MC group did not occur after 5 days of re-nutrition, and this effect was similar in the animals that received milk, PFM or BFS. This study was important to verify the safety of the re-nutrition diet used in our model, mainly the administration of the complete fermented milk due to the high number of bacteria (10^8 ^CFU/ml) it contains (probiotic bacteria and yoghurt starter cultures).

According to previous results where the administration of PFM to healthy mice increased the immune function and conferred beneficial changes to the intestinal microbiota [15], different bacterial populations were analyzed in our PEM model. It was reported that protein malnutrition disrupts the normal ecology of the microbiota with excessive growth of anaerobic microorganisms in the upper gastrointestinal tract [16-18]. The results obtained in the present work, after 5 days of re-nutrition showed significant increases for bifidobacteria when the mice received PFM or its BFS but not for milk re-nutrition (Figure 2C). This finding was important considering that bifidobacteria could confer a beneficial effect on the stability of the intestinal microbiota [19]. Allori et al [9] used an experimental murine model of severe malnutrition and showed that the equilibrium between aerobic and anaerobic strict bacteria in the gut microbiota was altered and that the ingestion of *L. casei *CRL 431 or conventional yoghurt was able to restore the gut microbiota. In contrast to these results, in our work the malnourished mice increased the number of total lactobacilli and even this increase was always significant compared to the well-nourished mice. After the re-nutrition period, malnourished mice re-nourished with any supplementary diet maintained the increases of total lactobacilli, which is important considering the beneficial properties attributes to *Lactobacillus *genus in the gastrointestinal tract. This difference and the lack of changes in the other bacterial populations after malnutrition period could be due to the fact that our model differs compared to the severe PEM model used previously by others.

Many beneficial effects especially on the immune system have been observed for bifidobacteria. They include: increased mucosal IgA production [20]; stimulation of phagocytic activity of mononuclear cells [21]; stimulation of natural killer cells activity [22]; increased lymphocyte responsiveness to oral and systemic challenge antigen [23,24].

In this sense, considering that the consumption of the PFM and its BFS increased this bacterial population, some immunological parameters were measured to analyze their influence on the regulation of the immune system and to demonstrate the usefulness of the PFM as oral adjuvant when it is administered to immune-deficient hosts.

The study of different immune cells showed that PFM administration was the only diet that increased IgA+ cells in the lamina propria of the small intestine after 5 days of re-nutrition, similar to those reported for the administration of the PFM in well nourished adult mice where IgA+ cells and IgA secretion to the intestinal fluid was increased after 5 days of PFM administration [12].

The gastrointestinal epithelium is covered by a protective mucus synthesized and secreted by goblet cells. The concept of the mucus layer functioning as a dynamic defensive barrier is suggested by studies showing altered mucus-layer in germ-free animals [25,26] and for consistent evidence of enhanced mucus secretion in response to intestinal microbes [27]. The number of goblet cells was not significantly affected by the non-severe PEM or the different re-nutrition conditions (Table 1). It is important to note that the period of consumption of the PFM re-nutrition did not increase goblet cells as was previously reported in well-nourished mice [28]; the effect of the PFM in our model was more effective on other immune cells which were severely affected by the restricted diet administered to induce malnutrition.

Dendritic cells are known to be essential immune cells in innate immunity and in the initiation of adaptive immunity. These cells capture and transfer information from the outside the body to the cells of the adaptive immune system. Macrophages are an important cell population for the innate immune response and might also be involved in the regulation of acquired immune responses [29]. It was reported that probiotic bacteria can exert their beneficial properties on the host immune system by activating these cells [30]. The marker F4/80 is present on the surface of a family of cells member of the mononuclear phagocyte system of mice. The expression of this antigen can be considered a specialized adaptive state rather than a separate lineage, which is higher in mature macrophages and its expression is required for regulatory T cell development [31].

With regards to the antigen recognized by 33D1 antibody, it is an inhibitory receptor and is present on a subpopulation of DC. The lack of this receptor might suggest a gain in function; however, DC recognized by 33D1 are more effective for antigen presentation on the class II major histocompatibility complex, than DC without this marker [32]; thus the antigen that binds 33D1 antibody on DC, may reflect their maturation state.

Macrophages and DC numbers decreased in the MC mice. After 5 days of re-nutrition, the three diets used increased the number of macrophages but only PFM and BFS administration increased DC in the lamina propria of the small intestine compared to MC group (Table 2). The results obtained for macrophages and DC were similar to those reported for well-nourished mice in a model where PFM was evaluated during nursing and after weaning until immune maturity [28].

Another immune cells studied in our model were the T lymphocytes. The results obtained did not show significant differences in the re-nourished mice compared to the MC group. The lack of effect on the number of these cells could be explained because it is know that the thymus suffer atrophy after malnutrition [33]. Maybe the period of time used for the re-nutrition was not enough to recover this organ or to stimulate a thymus independent production of T lymphocytes at the intestinal level from the cryptopatches [34].

To evaluate the functionality of the studied immune cells, even T cells which did not show numerical changes after re-nutrition, the number of various cytokines producing cells was analyzed (Table 3). IL-2 is a cytokine involved in the progression of T lymphocytes as a growth factor [35]. It is produced by Th1 lymphocytes and can also be produced by DC [36]. It was observed that re-nutrition increased the number of IL-2+ cells to values similar to the WC. This finding was important because IL-2 is an important nexus for the adaptive immunity.

TNF-α and IFN-γ are known pro-inflammatory cytokines. They are produced by activated cells and are able to activate other cells during inflammatory responses. However, it was recently demonstrated that these cytokines are more important in the crosstalk between immune cells than in the inflammatory response where IL-17 is involved [37], and these can be induced at the intestinal level after probiotic stimulation [38]. In our non-severe PEM model TNF-α+ and IFN-γ+ cell numbers were decreased in MC mice; the re-nutrition with milk and PFM increased their numbers in the small intestine. No effects were observed for these cytokines in mice that received BFS during re-nutrition period, showing the importance of the whole fermented product to activate the immune cells to produce cytokines. Similar results were obtained for IL-12+ cells. This cytokine plays a pivotal role in Th1 T cell differentiation and induces naive T cells to produce IFN-γ.

The study of IL-6 producing cells showed the importance of the soluble fraction (BFS), which was more effective to stimulate the production of this cytokine. This cytokine is also involved in the increased clonal expansion of IgA+ cells. PFM and milk administration did not increase the production of this cytokine which was diminished after malnutrition.

Considering the importance of maintaining the gut immune homeostasis by regulatory cytokines such as IL-10 and IL-4, these two cytokines were also analyzed. The results showed that IL-4 and IL-10 secreting cells were decreased in MC mice. The re-nutrition with PFM increased the production of these cytokines, being significant IL-4 increases. It is also important to remark that the re-nutrition with milk significantly increased the number of both IL-4+ and IL-10+ cells showing a higher down regulation than the response obtained with the PFM or its supernatant.

The increases observed for the number of macrophages in the intestinal tissues after re-nutrition led us to analyze the phagocytic activity of macrophages isolated from other sites such as spleen and peritoneum and due to the increased phagocytic activity observed after 5 days of re-nutrition with PFM and BFS (Figure 4), the adjuvant effect of these products on the systemic immune response was evaluated.

It was observed that malnutrition affected the systemic immune response against the OVA antigen (Figure 5). The three re-nutrition diets increased this specific response in the serum of the mice when they continued receiving the respective diet after antigen immunization, although the concentration of anti-OVA IgG was even high when the PFM was stopped. These results lead us to determine also the phagocytic activity of macrophages isolated from spleen and peritoneum after immunizations way of explaining the antibody increases (Figure 4). The results showed that the macrophage activity increased only previous to the immunization in the re-nourished mice. This observation indicates that the increase on the antigen presentation was important at this time point, the day of the immunization, before the adaptive response continued. However, the PFM administration was the only one that increased anti-OVA IgG in the serum of re-nourished mice even when they did not receive a specific diet after immunization. For milk and BFS, the continuous administration, after antigen immunization, was necessary to maintain a good antibody response.

The importance of the host immune system to confront different enteropathogenic infection and how probiotic administration can enhance this immune response exerting a protective effect was previously reported using animal models [14,39]. The changes observed in the immune response of mice re-nourished with PFM or its BFS led us to study their appliance in the defence against a common enterophatogenic infection. The study of *S. *Typhimurium infection in our model of malnutrition showed that only the animals that received PFM as a re-nutrition diet prior and after pathogen challenged decreased significantly the translocation of this bacterium to the different organs studied, compared to the others test groups. This finding agrees with previous results where the administration of the same PFM to well nourished adult or newborn mice (after weaning) exerted a protective effect against this pathogenic infection with improvement of the gut and systemic immune response in the mice administered PFM [40].

## Conclusion

The results obtained in the present work showed that in the non severe PEM, PFM was the re-nutrition diet most effective to improve the intestinal microbiota, increase the number and function of certain immune cells, especially from the innate immune response maintaining the intestinal homeostasis. This re-nutrition diet also stimulated the systemic immune response and could be suggested as adjuvant of the immune system in a malnourished host. The importance of the metabolites released during milk fermentation was also demonstrated through the analysis of the BFS, but the whole PFM showed the best effect on all the parameters studied, showing the importance of the presence of probiotic bacterium in order to exert a protective effect against *S*. Typhimurium infection.

We demonstrated that PFM could be useful to improve the gut and systemic immune response in an immunodeficiency model by malnutrition.

## Abbreviations

PEM: protein-energy malnutrition; WC: well-nourished control; MC: malnourished Control; M: milk; PFM: probiotic fermented milk; BFS: bacterial free supernatant; CFU: colony-forming unit; RCA: Reinforced clostridia agar; MRS: Mann-Rogosa-Sharp; PBS: phosphate buffered saline; OVA: chicken egg albumin; FITC: fluorescein isothiocyanate; DC: dendritic cells; TMB: 3,3',5,5' tetramethylbenzidine.

## Competing interests

This work was funded by grant between Danone - Argentina and CONICET. RW works in the Research and Development Division of DANONE-Argentina S.A. in Buenos Aires, participated in the conception of the study and in the revision of the manuscript. DANONE Argentina S.A. commercializes the fermented product investigated in this study. The rest of the authors declare that they have no competing interests.

## Authors' contributions

CMG, INN and AdMdL carried out the microbiological work and the animal studies. GP, EC and RW conceived of the study. CMG, AdMdL and GP designed the experiments. CMG, INN and AdMdL performed the statistical analyses and prepared the figures. CMG, INN, AdMdL and GP wrote the draft of the manuscript. EC, RW revised it for significant intellectual content. All authors read and approved the final version of the manuscript.

## Pre-publication history

The pre-publication history for this paper can be accessed here:

http://www.biomedcentral.com/1471-230X/11/64/prepub
